# Chemoresistance in Prostate Cancer Cells Is Regulated by miRNAs and Hedgehog Pathway

**DOI:** 10.1371/journal.pone.0040021

**Published:** 2012-06-29

**Authors:** Saurabh Singh, Deepak Chitkara, Reza Mehrazin, Stephen W. Behrman, Robert W. Wake, Ram I. Mahato

**Affiliations:** 1 Department of Pharmaceutical Sciences, University of Tennessee Health Science Center, Memphis, Tennessee, United States of America; 2 Department of Pharmaceutics, National Institute of Pharmaceutical Education and Research, SAS Nagar (Mohali), Punjab, India; 3 Department of Urology, University of Tennessee Health Science Center, Memphis, Tennessee, United States of America; 4 Department of Surgery, University of Tennessee Health Science Center, Memphis, Tennessee, United States of America; Institute of Hepatology London, United Kingdom

## Abstract

Many prostate cancers relapse due to the generation of chemoresistance rendering first-line treatment drugs like paclitaxel (PTX) ineffective. The present study aims to determine the role of miRNAs and Hedgehog (Hh) pathway in chemoresistant prostate cancer and to evaluate the combination therapy using Hh inhibitor cyclopamine (CYA). Studies were conducted on PTX resistant DU145-TXR and PC3-TXR cell lines and clinical prostate tissues. Drug sensitivity and apoptosis assays showed significantly improved cytotoxicity with combination of PTX and CYA. To distinguish the presence of cancer stem cell like side populations (SP), Hoechst 33342 flow cytometry method was used. PTX resistant DU145 and PC3 cells, as well as human prostate cancer tissue possess a distinct SP fraction. Nearly 75% of the SP cells are in the G0/G1 phase compared to 62% for non-SP cells and have higher expression of stem cell markers as well. SP cell fraction was increased following PTX monotherapy and treatment with CYA or CYA plus PTX effectively reduced their numbers suggesting the effectiveness of combination therapy. SP fraction cells were allowed to differentiate and reanalyzed by Hoechst staining and gene expression analysis. Post differentiation, SP cells constitute 15.8% of total viable cells which decreases to 0.6% on treatment with CYA. The expression levels of P-gp efflux protein were also significantly decreased on treatment with PTX and CYA combination. MicroRNA profiling of DU145-TXR and PC3-TXR cells and prostate cancer tissue from the patients showed decreased expression of tumor suppressor miRNAs such as miR34a and miR200c. Treatment with PTX and CYA combination restored the expression of miR200c and 34a, confirming their role in modulating chemoresistance. We have shown that supplementing mitotic stabilizer drugs such as PTX with Hh-inhibitor CYA can reverse PTX chemoresistance and eliminate SP fraction in androgen independent, metastatic prostate cancer cell lines.

## Introduction

Prostate cancer is the second leading cause of cancer related death in men in the United States [1]. While anti-androgen therapy is currently the first line of treatment for patients diagnosed with prostate cancers, most patients will eventually develop the androgen-independent form of prostate cancers which is highly metastatic and has poor prognosis [2]. Microtubule stabilizers such as PTX are effective in treating patients diagnosed with androgen-independent prostate cancer [3]. While clinical trials have proven the initial efficacy of taxanes in increasing survival in prostate cancer patients [4], there are currently few effective approaches for treating chemoresistant prostate cancers.

Most tumors are heterogeneous and are composed of bulk and tumor initiating cells (TICs) with the latter forming a distinct subpopulation in many cancers. TICs are often referred to as cancer stem cells (CSCs) and are responsible for tumor initiation, self-renewal, and chemoresistance [5], [6]. Many prostate cancers relapse due to the presence of highly chemoresistant tumor initiating/cancer stem cells [7], [8]. Chemoresistance to anticancer drugs including PTX, by these cells may be contributed by drug-efflux pumps which can efficiently remove lipophilic molecules, including hydrophobic anticancer drugs. This inherent property of chemoresistant cells is used for identification and isolation of a side population (SP), which are a type of cancer stem cells. The SP fraction, initially identified by Goodell, is a small subpopulation of cells with enriched stem cell activity and are known to demonstrate distinctively low levels of Hoechst 33342 dye staining [9]. SP fraction cells have been shown to be insensitive to various chemotherapeutic drugs [10] owing to their ability in effluxing chemotherapy drugs (and lipophilic dyes such as Hoechst 33342) due to the high expression of ATP-binding cassette family, such as MDR1 (P-glycoprotein) and ABCG2 [11]. Chemoresistant SP cells will survive and sustain their clonogenicity during initial exposure to cytostatic drugs, thereby allowing disease recurrence when therapy is withdrawn. These subsets of CSCs are thus considered a viable target for improved therapeutic intervention and preventing chemoresistance and cancer relapse.

The development of chemoresistance through an increase in the number of cancer stem like cells, including SP fractions has been attributed to alterations at the level of microRNAs (miRNAs) in various cancer types. These non-coding RNA molecules can act as oncogenes as well as tumor suppressor [12], [13], [14]. Dysregulation of miRNAs has been implicated in tumorigenesis and drug resistance as well. Recent work by Cochrane et al. has identified miRNAs involved in modulating chemoresistance in several cancers [15].

In our present study, we hypothesized that chemoresistance to PTX in metastatic prostate cancer cells could be due to the altered miRNA expression in these cells and that the combination of antimitotic drug with another small molecule that inhibits CSCs is likely to be effective in not only reverting chemoresistance by suppressing CSCs but also target miRNAs involved in chemoresistance. Thus, while failure of traditional chemotherapy is due to a failure to destroy CSCs/SP fractions, a combinatorial approach is likely to yield better results since the CSC-inhibitor will kill pluripotent cancer cells and will allow the antimitotic drug (in this case PTX) to attack bulk tumor cells. Towards this end, we have combined PTX with cyclopamine (CYA), a natural steroidal alkaloid which inhibits the Hedgehog (Hh) pathway resulting in decreased proliferation and increased apoptosis [16]. In recent years, the Hh signaling pathway has been implicated in the development and spread of prostate cancer [17], [18]. Evidence has also indicated that Hh signaling supports androgen signaling and androgen-independent growth in prostate cancer cells in a low androgen environment [19]. Inhibition of Hh-pathway results in downregulation of genes involved in stem cell self-renewal as well as regression of prostate tumor without relapse [20]. Combination of docetaxel with CYA and epidermal growth factor receptor (EGFR) inhibitor gefitinib induced greater antiproliferative and apoptotic effects on SP cell fractions isolated from metastatic prostate cancer cells than individual drugs [21]. We have recently demonstrated that adding EGFR-inhibitor lapatinib can enhance the effectiveness of PTX in inducing apoptosis in a paclitaxel-resistant, androgen-independent metastatic prostate cancer cells line DU145-TXR, both *in vitro* as well as in xenograft tumors [22].

To understand the phenomenon of chemoresistance, in the present study we have used androgen independent (AI) metastatic prostate cancer cell lines DU145 and PC3 and their PTX-resistant versions, DU145-TXR and PC3-TXR, respectively. We have shown that PTX resistance of prostate cancer cells may be modulated at the level of miRNAs. We further demonstrate that combination therapy with CYA and PTX can effectively reduce cell viability, decrease SP-cell fraction at doses far lower than that used for CYA monotherapy and impact miRNAs putatively involved in modulating chemoresistance. Our data indicates that combination therapy involving supplementation of PTX with Hh-inhibitors can target specific miRNAs and cancer stem-cell like SP cell populations at doses that are effective in combination but not in monotherapy. This approach may represent a better approach in preventing metastasis and relapse in refractory prostate cancer since it is less likely to be toxic and will present with far fewer side effects for the patient, ensuring better compliance and reducing the chances of a recurrence.

## Materials and Methods

### Materials

PTX and CYA were purchased from LC Labs (Woburn, MA). SYBR Green real-time PCR master mix and reverse transcription reagents were purchased from Applied Biosystems (Foster city, CA). Goat anti-rabbit P-gp antibody and corresponding secondary antibody was purchased from Santa Cruz Biotechnology (Santa Cruz, CA). All other chemicals were obtained from Sigma-Aldrich (St. Louis, MO) and used as received, unless stated otherwise.

### Cell lines

The human metastatic prostate cancer cell lines DU145 and PC3 and their PTX resistant versions DU145-TXR and PC3-TXR were a kind gift of Prof. Evan T. Keller (University of Michigan). All cell lines were maintained in RPMI culture media supplemented with 1% penicillin/ streptomycin and 10% fetal bovine serum (FBS) (Gibco) in a humidified incubator containing 5% CO_2_ at 37°C as described earlier [22].

### Human Prostate Tissue

Human prostate tissue (cancerous and benign) were obtained from the Veterans Affairs (VA) Hospital, Memphis, TN following established protocols and in accordance with the informed consent waiver provided by the Institutional Review Board (IRB) at UTHSC and at the VA Hospital. Prostate tissue was taken using an 18-gauge core needle biopsy gun and a portion of this tissue was rinsed and either flash frozen in liquid nitrogen and then stored at −80°C or placed in cold serum-free RPMI media containing antibiotics for preparing single cell suspensions. Tissues were classified as malignant or benign based on the diagnosis made by a pathologist.

### Drug sensitivity and apoptosis assays in DU145-TXRCells

To determine the extent of cellular apoptosis following drug treatments, DU145-TXR cells were plated into 6-well plates (7.5×10^5^ cells/well). After 24 h, the media was removed and fresh media containing varying concentrations of PTX, CYA or their combinations were added. The cells were then stained with Annexin-V and Propidium iodide (PI) using the Vybrant Apoptosis Assay Kit as per the manufacturer's protocol (Molecular Probes). Briefly, cells were trypsinized, washed twice with cold PBS and pelleted by centrifugation at 800 rpm for 5 min. The pellets were resuspended in 100 μl of 1X Annexin binding buffer and 5 μl fluorescein isothiocyanate (FITC)-Annexin-V. Propidium iodide (100 μg/ml) was added to each 100 μl of cell suspension. The stained cells were immediately analyzed by flow cytometry.

DU145-TXR cells were also used to determine the cell growth inhibition ability of PTX and CYA. Cells (5×10^3^/well) were seeded in 96 well cell culture plates and incubated for 24 h to allow cell attachment. Media was then replaced with fresh media containing PTX (0.5/1 µM) or CYA (10/25 µM) or combination (PTX 0.5 µM and CYA 10µM) and incubated further for 48 h at 37°C/5% CO_2_. Cell viability was then assessed by MTT assay. For this, media was removed and cells were washed with PBS and 200 µl of fresh media containing 3-(4,5-dimethyl-thiazol-2-yl)-2, 5-diphenyl tetrazolium bromide (MTT) (0.5 mg/ml) was added followed by incubation at 37°C/5% CO_2_ for 4 h. After 4 h, media was removed and formed formazan crystals were dissolved in 200 µl DMSO and absorbance was measured at 560 nm. Cell viability was calculated using the following formula:
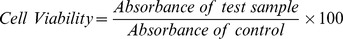



DMSO was used to solubilize PTX and CYA and DMSO controls were included in all experiments.

### Side Population analysis and cell sorting by FACS

Side population analysis in DU14-TXR and PC3-TXR cell lines was performed using Hoechst 33342 flow cytometry method. In brief, adherent cells were trypsinized and washed with phosphate buffered saline (PBS). Cells (1×10^6^ cell/ml) were then suspended in RPMI media supplemented with 2% FBS and 1 mM HEPES buffer with or without drug solutions (Verapamil, PTX or PTX+CYA). Hoechst 33342 (5 µl; 1 mg/ml) dye was then added followed by incubation for 90 min at 37°C. Cells were recovered by centrifugation and washed several times with PBS to remove unbound dye and finally suspended in ice cold PBS containing 2% FBS. SP fraction in clinical samples was analyzed as described above with additional steps. Briefly, freshly resected prostate tissue was rinsed, mechanically minced and digested for 4 h at 37°C with 100 U/ml collagenase IV (Worthington Biologicals) in serum free RPMI. The tissue was frequently pipetted with a 5-ml serological pipette and at the end of incubation the digest was passed through an 18.5- gauge needle, centrifuged briefly and the supernatant sieved through a 100 µm cell strainer to obtain single cell suspension. Diluted single cell suspensions were then passed once through 40 µm mesh filter, their viability assessed by trypan blue staining and kept on ice until analyzed.

**Figure 1:**
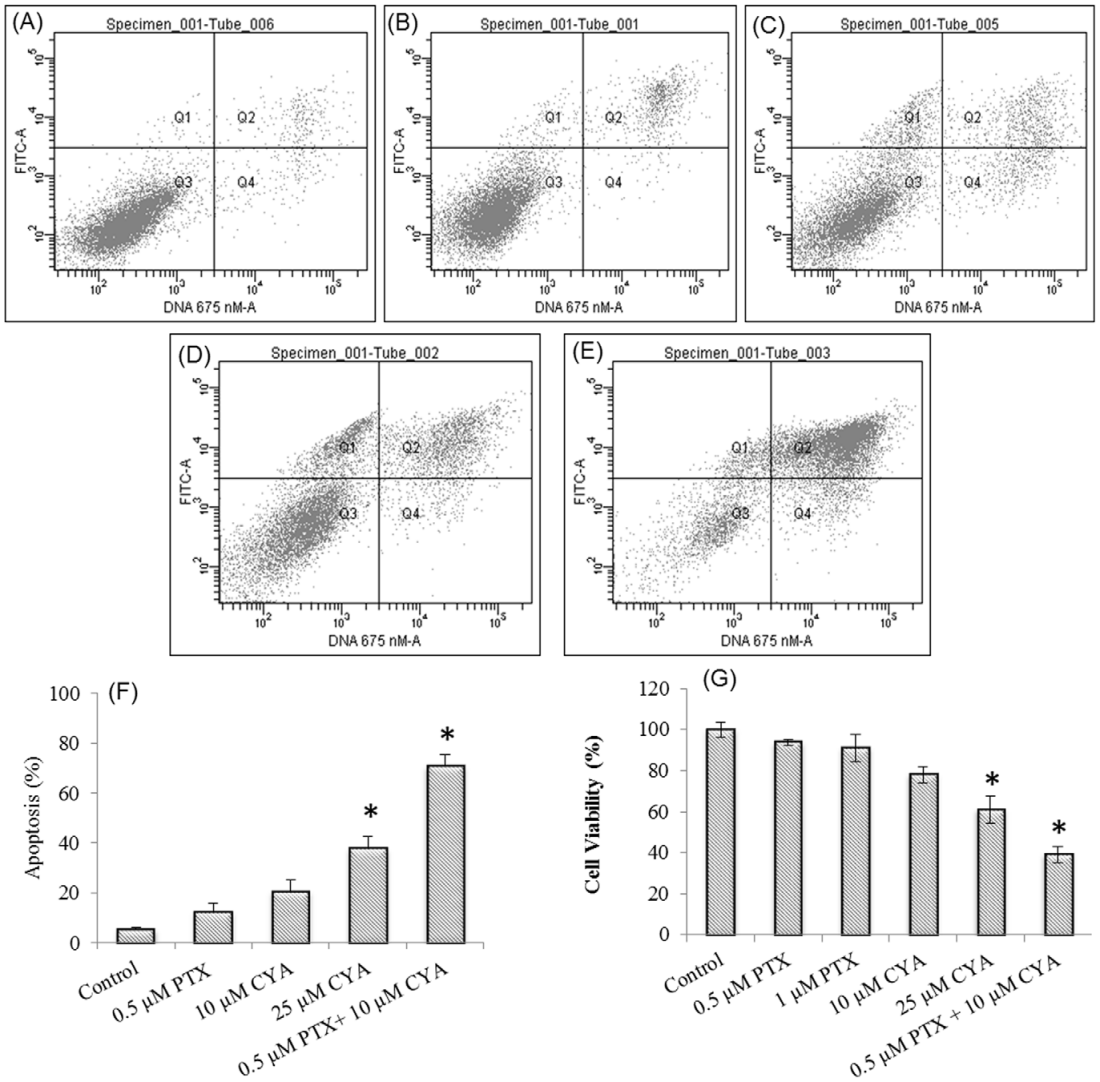
Effect of PTX and CYA combination on viability of PTX resistant DU145-TXR cells. Cells grown in 6-well plates were treated with A) 0.3% DMSO, B) 0.5 µM PTX C) 10 µM CYA, D) 25 µM CYA, E) 0.5 µM PTX +10 µM CYA for 48h and F) in DU145-TXR cells after different drug treatments. Subsequently, cells were trypsinized, washed with PBS and stained with Annexin V-FITC and PI before apoptotic analysis by flow cytometry. A–E are representative plots from three individual experiments. Data in panel F is the quantitation of % cell death and represents mean ± SD (n = 3). *p<0.05 vs. control. For MTT assay (Fig. 1G), cells grown in 96 well plate were treated with indicated concentration of drugs for 48 h. Subsequently, MTT reagent in PBS was added and incubation was carried out for another 4 h. The resulting formazan product was solubilized in DMSO and the color intensity was determined using a plate reader. A statistically significant difference (* *p* value <0.05) was observed when combination of 0.5 µM PTX and 10 µM CYA was used. Cell Viability = A_test_/A_control_X100. Data are the means±SD (n = 4). PTX, Paclitaxel; CYA, Cyclopamine.

### Cell Cycle analysis

Flow cytometry was used to determine the percentage of cells in different growth cycles. Cells (5×10^5^) obtained after sorting were washed with PBS and fixed with ethanol (70%) at 4°C overnight followed by treatment with RNAse (1 mg/ml) and stained with PI (10 µg/ml). Percentage of cells in different cell cycle phases was then determined.

**Figure 2:**
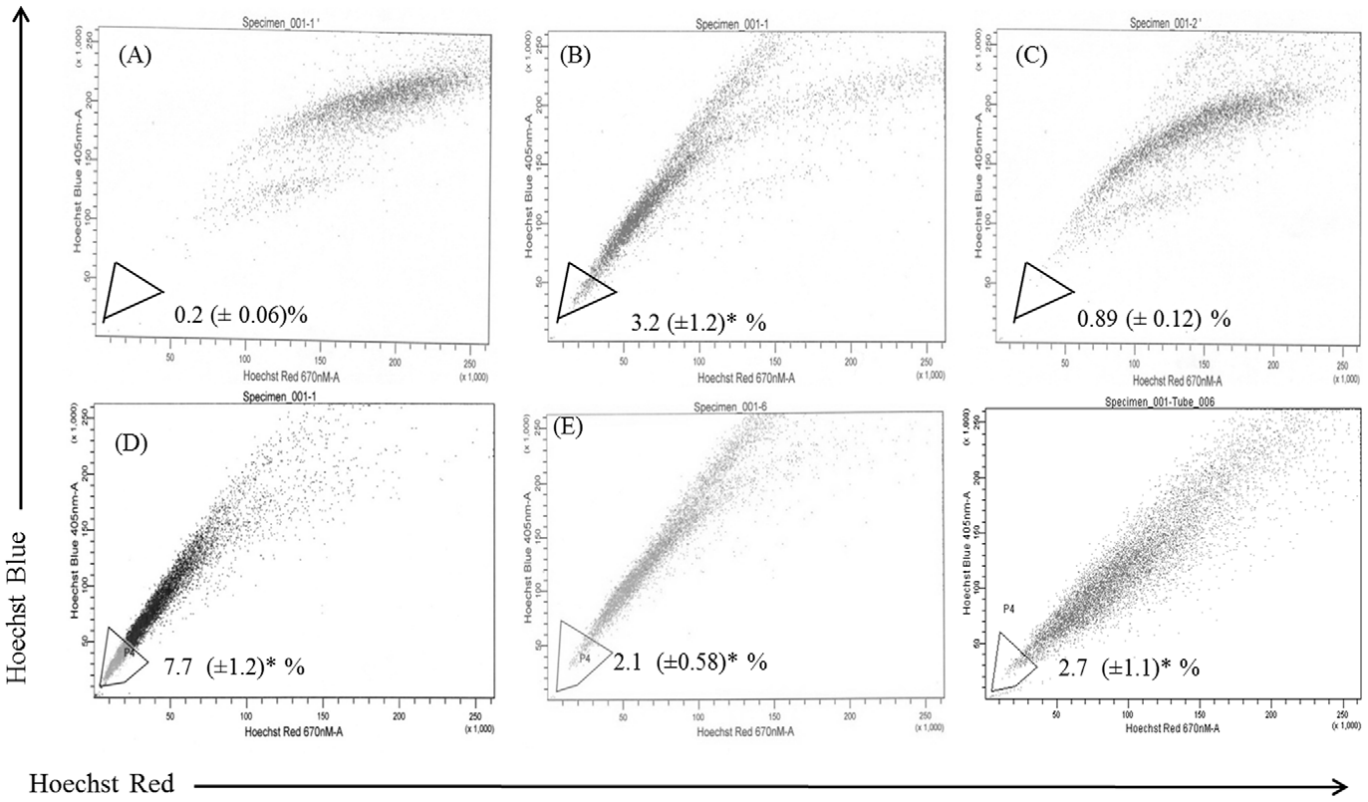
Analysis of side population (SP) fraction in PTX resistant DU145 TXR cells after treatment with PTX and CYA. A) DU145 cells, B) DU145-TXR cells, C) DU145-TXR cells treated with verapamil (10 µM, 90 min), D) DU145-TXR cells treated with PTX (1 µM, 12 h), E) DU145-TXR cells treated with CYA (20 µM, 12 h), F) DU145-TXR cells treated with CYA and PTX (20 µM and 0.5µM, respectively, 12 h). Verapamil was used to gate the SP fraction in all panels and shown as the percentage of the whole viable cell population. Numerical values indicated are the mean±SD of three individual experiments. * *p*<0.05 vs control DU145 cells in (A). PTX, Paclitaxel; CYA, Cyclopamine.

**Figure 3:**
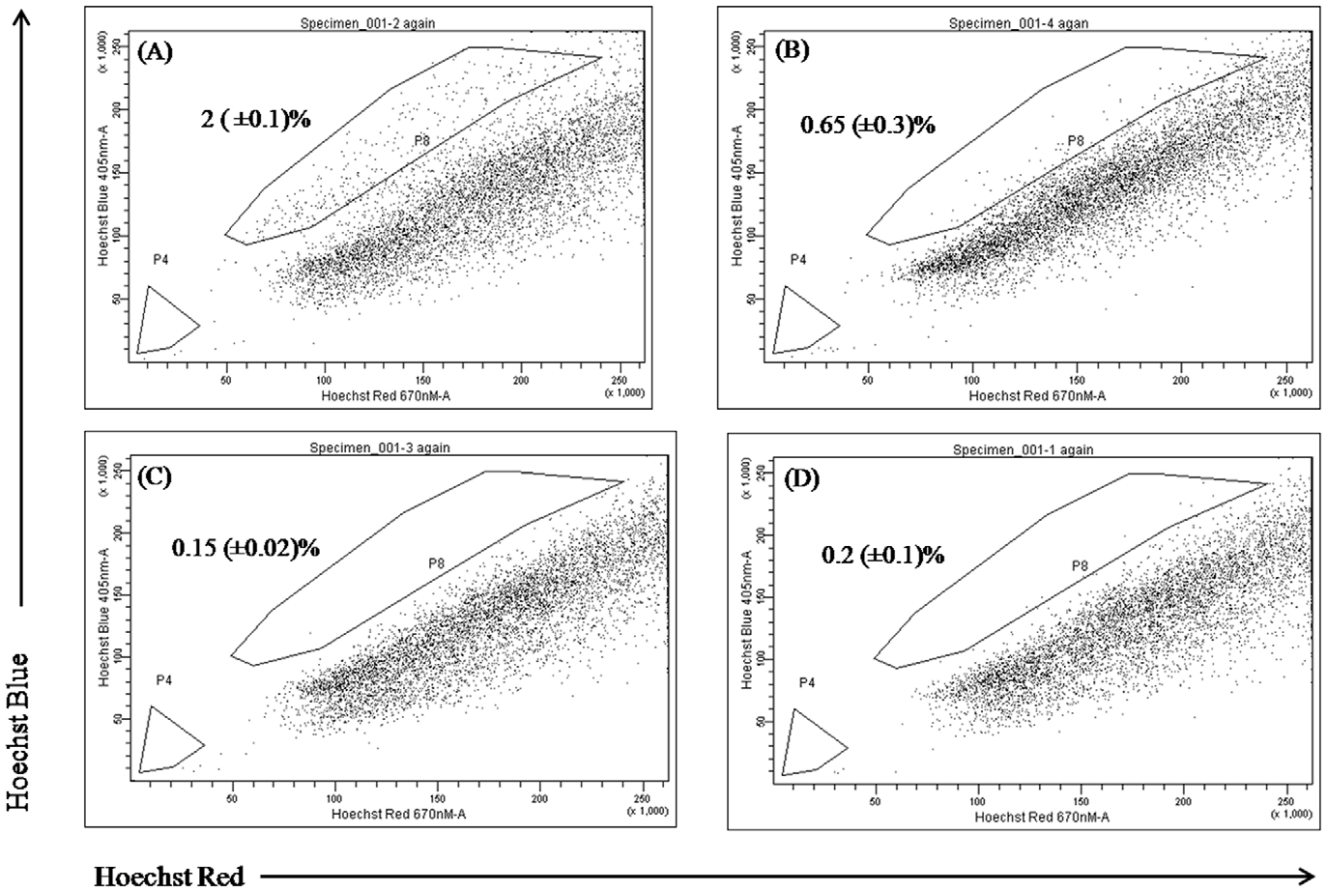
Analysis of side population (SP) fraction in PTX resistant PC3-TXR cells after treatment with PTX and CYA. A) PC3-TXR cells, B) Cells treated with verapamil (10 µM, 90 min), C) PC3-TXR cells treated with CYA (20 µM, 12 h) or, D) CYA and PTX (20 µM and 0.5µM, respectively, 12 h). The SP fraction, which was eliminated by treatment with verapamil, was gated (P8) in all panels and shown as the percentage of the whole viable cell population. Numerical values indicated are the mean±SD of three individual experiments. * *p*<0.05 vs control DU 145 cells (A). PTX, Paclitaxel; CYA, Cyclopamine.

**Figure 4:**
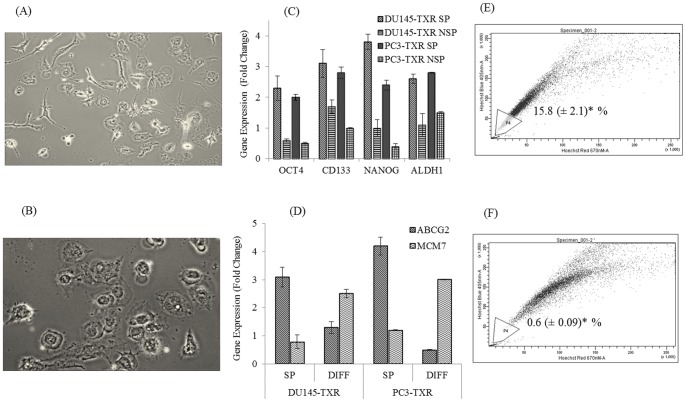
Post differentiation fate of side population fractions from DU145-TXR cells. After flow sorting, pure SP cells were plated and allowed to differentiate for 2-weeks. Representative photomicrographs of SP (A) and NSP cells (B) are shown with more SP cells possessing a fibroblastic, elongated phenotype compared to NSP. Real time RT-PCR was used to confirm higher expression of stem cell markers OCT 4 and NANOG and cancer stem cell markers CD133 and ALDH1 (C). Similar method was used to show higher expression of pluripotency and efflux marker ABCG2 and lower expression of MCM7 transcripts in initial SP cells (SP), compared to mixed populations post-differentiation (DIFF) where decreased ABCG2 and increased MCM7 levels were observed (D). One set of cells was also treated with 25 µM CYA for 48h. Subsequently, cells were trypsinized and re-stained with Hoechst dye and analyzed by flow cytometry. Post-differentiation, cells derived from flow sorted SP cells had higher percentage of SP cell fractions than obtained previously from non-sorted DU145-TXR cells. CYA treatment significantly reduced (*p*<0.001 vs. control) the percentage of SP fraction cells from 15.8 (±2.1) % (Panel E) to 0.6±(0.09) % (Panel F). Representative dot plots from three individual experiments are provided. Data represents mean ± SD (n = 3).

**Table 1:** Cell Cycle distribution of SP and non-SP Cells following cell sorting.

DU 145 TXR cells	Cell Cycle Distribution (%)		
	G0-G1	S	G2-M
SP	71.5±1.5*	21±2.4*	7.5±2.7
Non-SP	62.08±2.3	30±1.6	7.92±3.2

SP and non-SP fractions were fixed overnight in ice cold 70% ethanol as indicated in ‘Methods’. Subsequently, they were washed with PBS and stained with a solution of propidium iodide (PI) and RNAase A before flow cytometry. A statistically significant difference was observed in the cell cycle distribution. *p<0.05 vs. Non-SP cells.

### In vitro differentiation study

Ability of SP cells to differentiate was determined by culturing the pure cell fractions in a 6 well plate for 14 days post sorting. SP-fraction cells from DU145-TXR and PC-TXR (1×10^5^/well) were seeded into 6 well plate and allowed to grow in RPMI culture media supplemented with 10% FBS. After 14 days, Hoechst staining and SP analysis was done on treated or untreated cell populations as described above. Gene expression analysis was also carried out on SP and non-SP cells both before and after-differentiation.

**Figure 5:**
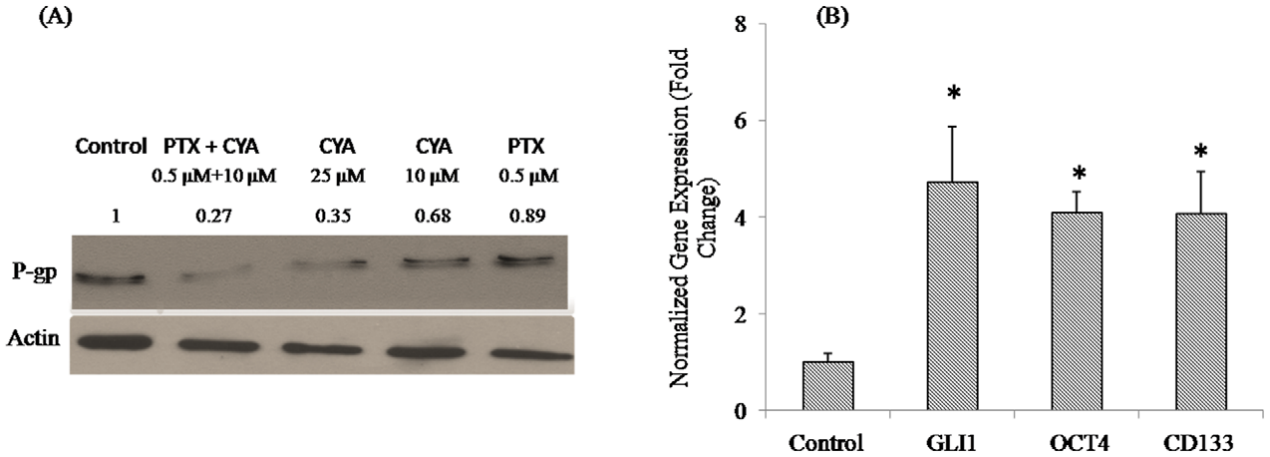
Effect of PTX and CYA on P-gp expression in DU145-TXR cells. Following treatment, with various drugs as described, total protein was extracted and separated by SDS-PAGE before probing with P-gp antibody. Actin was used as a loading control. A combination of 0.5 µM PTX and 10 µM CYA was more effective in downregulating P-gp expression in drug-resistant prostate cancer cells than monotherapy with either CYA or PTX at 10 and 0.5 µM concentration. P-gp downregulation with 25 µM CYA was nearly similar to that obtained by combination therapy. (B) Expression of Hh pathway and stem cell marker genes in DU145-TXR cells. Total RNA was extracted from cells and reverse transcribed to cDNA. Real time RT-PCR was carried out using SYBR Green chemistry and Ct values thus obtained were used to calculate the fold change. Drug resistant DU145-TXR cells have higher expression of all three genes tested. PTX sensitive DU145 cells were used as control and gene expression values for DU145-TXR cells were normalized with respect to the control values. *p<0.05 vs. control.

### Western blot analysis

Following treatment, DU145 TXR cells were lysed using RIPA buffer and total protein concentration was determined using Bio-Rad RC DC protein assay kit (Hercules, CA). SDS-PAGE was then performed to resolve the proteins which were then transferred to Immobilon polyvinylidene fluoride (PVDF) membrane using iBlot dry blotting system (Invitrogen, Carlsbad, CA). Blocking was done using 5% non-fat dry milk in 1X PBST (PBS containing 0.05% Tween-20) for 1 h at room temperature. Membranes were then incubated with primary antibody for 16 h at 4°C. Actin was used as the loading control and target protein was detected by enhanced chemiluminescence (ECL) detection kit (GE Healthcare Life Sciences, PA).

**Figure 6:**
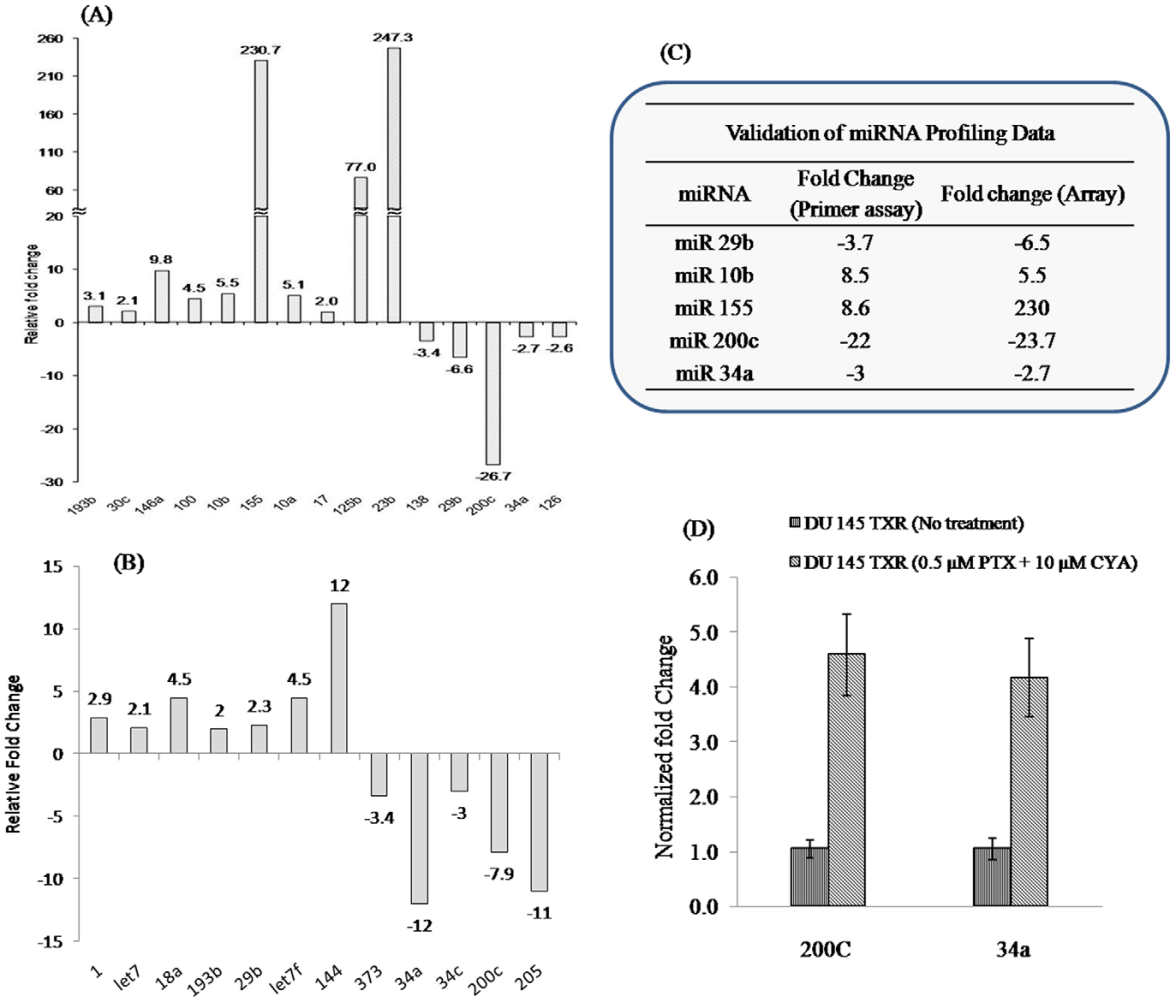
miRNA profiling of prostate cancer cells and effect of combination therapy on miRNA expression. Total RNA including miRNAs was isolated from DU145-TXR and DU145 cells (A) or PC3 and PC3-TXR cells (B) using miRNEasy RNA isolation kit. SYBR Green based pathway-focused miScript miRNA PCR Array (Qiagen, MD) was used for miRNA profiling studies. The plates were run on a Roche Light Cycler 480® instrument and the expression of individual miRNAs was analyzed using the obtained C_p_ values and the ΔΔCt method. Table in the insert (C) confirms validation of miRNA profiling data by miScript primer assay. Validation of miRNA profiling data was done by a SYBR Green based real time RT-PCR assay of selected miRNAs. As a normalizer, SNORD6 was used as a housekeeping miRNA. (D) Efficacy of combination therapy on restoration of miR 200 c and 34a was determined by treating DU145-TXR cells with PTX (0.5 µM) and CYA (10 µM)combination for 48 h after which total RNA was extracted, converted to cDNA and used as template for miScript primer assay for determining expression of miRNAs 200c and 34a. Untreated DU145-TXR cells were used as control for calculating fold change after a SYBR Green real time RT-PCR assay. Data represents mean ± SD (n = 3). * *p*<0.05 vs. untreated control.

### Real time RT-PCR

Total RNA was extracted from sorted and unsorted prostate cancer cells using RNeasy RNA isolation kit (Qiagen, MD), followed by determination of its quality by Nanodrop 2000 instrument. It was then reverse transcribed into cDNA template as described before [23]. cDNA (100 ng) was then amplified by real-time PCR using SYBR Green dye universal master mix on a Light Cycler 480 instrument (Roche, Indianapolis, IN) using the primers for genes of interest for forty cycles Relative amount of mRNA compared to S19 (housekeeping gene) level was calculated using Crossing point (Cp) values and scaled relative to control samples set at a value of 1. Results for gene expression in experimental samples were plotted compared with the control.

**Figure 7:**
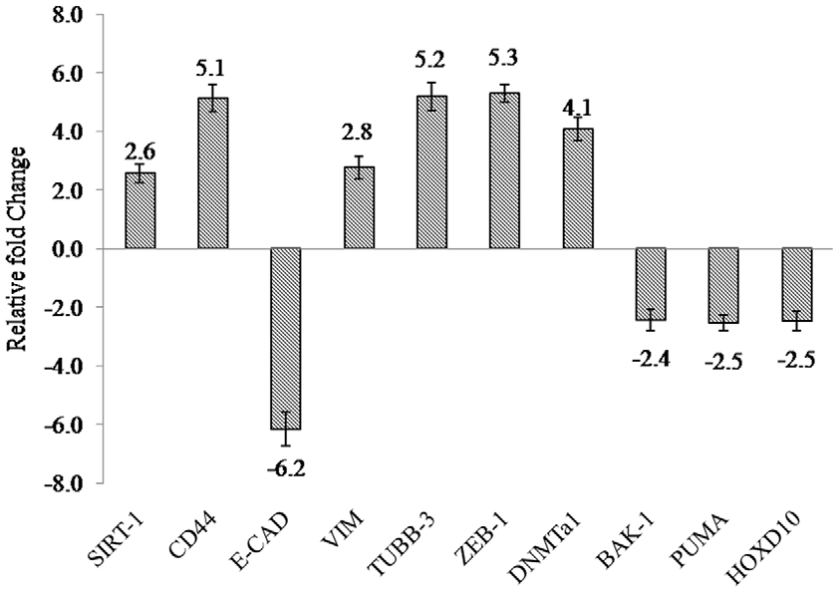
Real time RT-PCR identification of gene targets of miRNA in DU145-TXR Cells. Several genes involved in cancer related biological processes and known targets of differentially expressed miRNAs in our study are altered in PTX resistant DU145 TXR cells. Following RNA extraction and SYBR Green based real time RT-PCR using specific gene primers, Cp values were calculated and resistant DU145 cells were used to normalize the expression of individual genes. Data represents the mean ± SD (n = 3).

### MicroRNA (miRNA) profiling and data validation

Total RNA that includes small non-coding miRNA was isolated from untreated and drug-treated DU145-TXR and DU145 cells or PC3 and PC3-TXR cells using miRNEasy RNA isolation kit (Qiagen MD) following manufacturer's instructions. The same reagents were used to isolate total RNA from human prostate tissue. Briefly, flash frozen tissue was suspended in extraction buffer and subjected to careful disintegration using a hand-held electric homogenizer for 30s at a low-to-middle speed setting. The homogenate was centrifuged for 3 minutes at 4°C and the supernatant was used to extract total RNA. Post-isolation, RNA quality was determined using a Nanodrop 2000 instrument.

**Figure 8:**
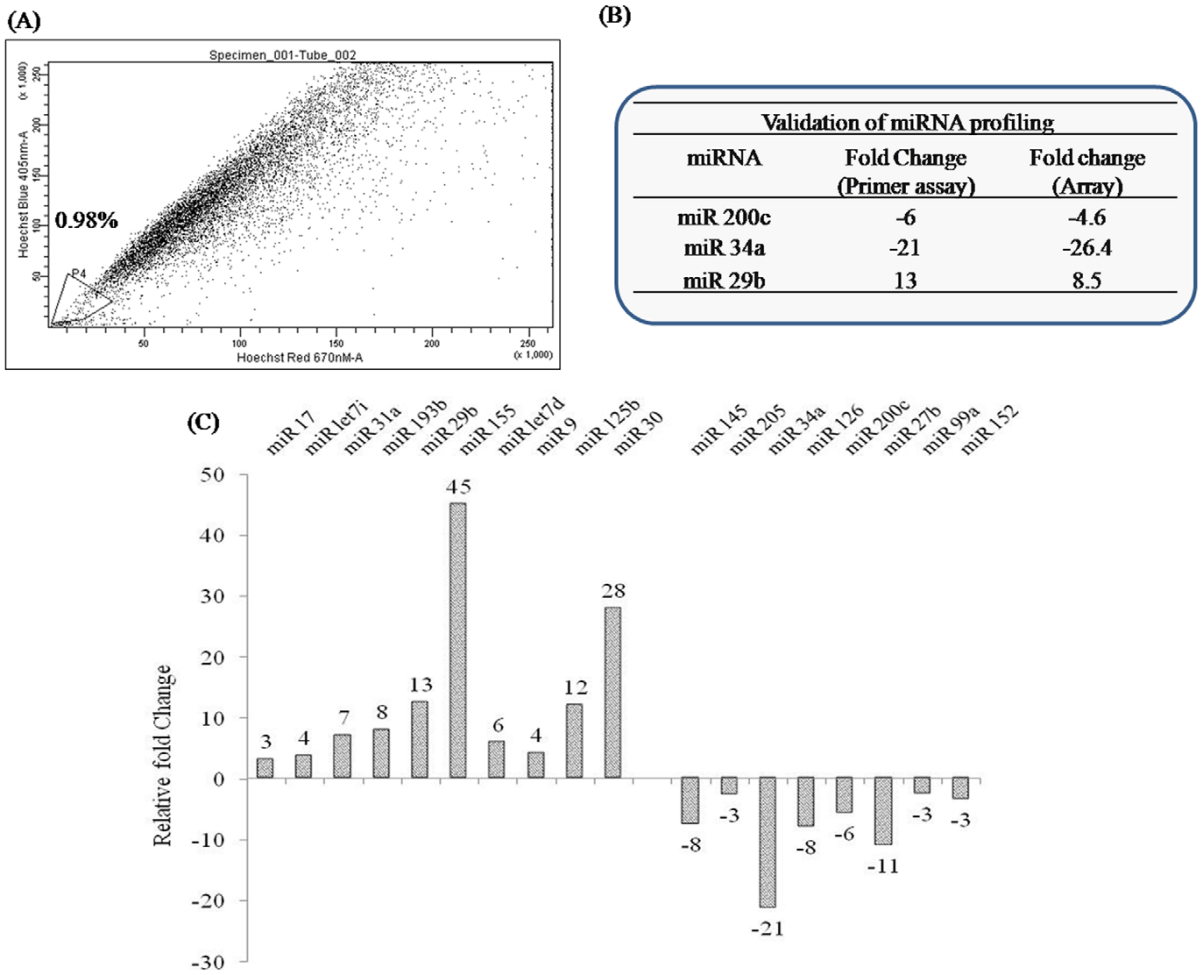
SP fraction analysis and miRNA profiling of clinical prostate tissues. (A) Human prostate cancer tissue was converted to single cell suspensions as described in ‘Methods’. Cells were stained with Hoechst dye and analyzed as described previously. Nearly 1% of total viable cell population was gated as the SP fraction. (B) Total RNA was isolated from another set of human prostate tissues (cancer and benign) using miRNEasy RNA isolation kit. SYBR Green based pathway-focused miScript miRNA PCR Array (Qiagen, MD) was used for miRNA profiling studies. The plates were run on a Roche Light Cycler 480® instrument and the expression of individual miRNAs was analyzed using the obtained C_t_ values and the ΔΔCt method. The fold changes in the tumor tissues were normalized with respect to the benign prostate tissue. (C) Table in the insert confirms validation of miRNA profiling data by miScript primer assay. Validation of miRNA profiling data was done by RT-PCR estimation of selected miRNAs200c, 34a and 29b. SNORD6 was used as a housekeeping miRNA for data normalization.

For miRNA profiling studies, SYBR green based pathway-focused miScript miRNA PCR Array (Qiagen, MD) was used. The cancer pathway array (catalog number 102ZF) allows the simultaneous detection of 84miRNAs previously identified in human cancers, as well as appropriate housekeeping assays and RNA quality controls. The assay was performed according to the manufacturer's protocol. Three hundred nanograms (ng) of total RNA were converted to cDNA using miScript II RT Kit. Diluted cDNA was mixed with universal primer and SYBR Green dye and added to the wells of 96-well plates containing lyophilized primer. The plates were run on a Roche Light Cycler 480®instrument and the expression of individual miRNAs was analyzed using the obtained C_t_ values. The fold change for each miRNA was calculated by plugging the C_p_ values into the manufacturer's web-based software. DU145 cells or PC3 cells, as well as benign prostate samples were considered as ‘controls’ in order to calculate the fold change in DU145-TXR and PC3-TXR cells and cancerous prostate tissue, respectively. Endogenous controls, RT negative and positive controls, and genomic DNA contamination controls were also tested for each array.

**Figure 9:**
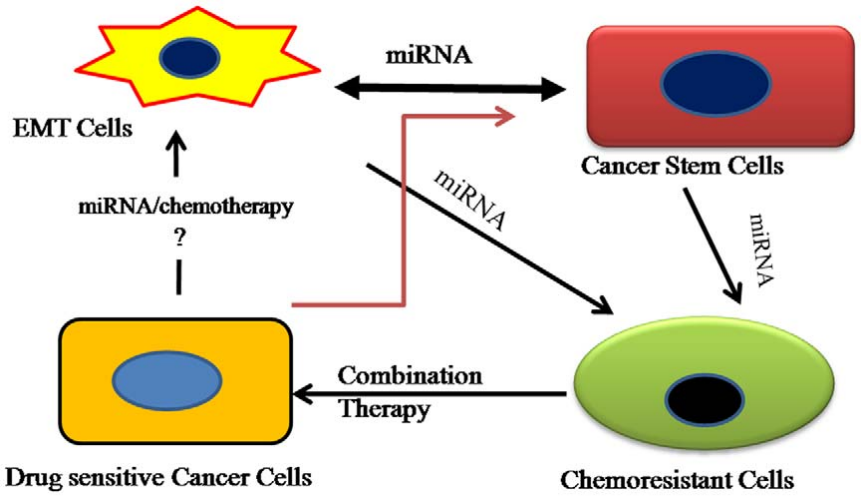
Interrelationship between EMT, cancer stem cells, miRNA and chemoresistance. Significant interrelationship exists between chemoresistance, epithelial to mesenchymal transition and metastasis, which adversely impacts treatment outcomes. Chemoresistance, a key feature of CSCs and cells undergoing EMT, is regulated by the dysregulation of miRNAs. Our proposed combination therapy with PTX and CYA simultaneously targets CSCs and bulk cancer cells, reverses EMT and restores expression of miRNAs altered during generation of chemoresistance and is a viable strategy for treating drug-resistant prostate cancer.

Validation of miRNA profiling data was done by real-time PCR estimation of selected miRNAs. For each of the selected miRNA, a miScript PCR primer was purchased from Qiagen. This assay targets only mature miRNAs, not their precursors. As a normalizer, SNORD6 was used as a housekeeping miRNA. Briefly, diluted cDNA used for miRNA profiling was used as a template in the presence of SYBR Green dye, universal primer and miScript primer. The plate was run as described above and fold changes in miRNA expression were calculated using the C_t_ value of the normalizer control. A similar approach was used to measure expression of miR200c and miR 34a in DU 145-TXR cells treated with 0.5 µM PTX+10 µM CYA. After treatment, total RNA was isolated, reverse transcribed to cDNA and used to measure miRNA expression as described above.

### Statistical Analysis

All values in the figures and text were expressed as the mean ± SD. The results were analyzed and individual group means were compared with Student's unpaired *t*-test. A *p* value of at least 0.05 was considered significant and is indicated by an asterisk (*).

## Results

### Combination of CYA and PTX reduces cell viability and enhances apoptosis in drug-resistant prostate cancer cells

The ability of combination chemotherapy to inhibit the growth of PTX resistant prostate cancer cell line was assessed by cell viability and apoptosis assay. It was observed that combination of PTX and CYA significantly (*p* value<0.05) decreases cell viability to 40% as compared to either PTX or CYA alone (Fig. 1, panels A–F). Similar results were obtained in Annexin V cell death assay wherein combination therapy results in a significantly higher cell death as compared to single agent chemotherapy (Fig. 1, G). While a combination of PTX and CYA resulted in nearly 70% of cells dying, the percent cell death observed with PTX or CYA monotherapy was 15% and 25% respectively. However, treatment with 25 µM CYA was significantly more effective than control and resulted in nearly 40% cell death.

### Side Population fractions exist in PTX -resistant prostate cancer cells and have unique gene expression

Figures 2 and 3 show the SP analysis of prostate cancer cell lines DU145-TXR and PC3-TXR, respectively as well as the effect of treatment with PTX and CYA. Control DU145 cells have tiny amounts of SP (0.2%, Fig. 2A) while PTX resistant cell line DU145-TXR has ∼3.2% of SP cells (Fig. 2B) as indicated by Hoechst staining (*p*<0.05 compared to DU 145 cells). In case of PC3-TXR cells, nearly 2% of viable cells were gated as SP (Fig. 3A). Verapamil, a known suppressor of efflux pumps was used in these studies as a control to set up the gates in the FACS dot plots. As seen in Figures. 2C and 3B, verapamil treatment significantly reduced SP fractions in DU145-TXR cells to 0.89% and to 0.6% in PC3-TXR cells. This data is in agreement with the use of verapamil as a control drug for identifying and gating Hoechst-light cells in various cancer cells, including prostate cancer. While treatment with 1 µM PTX for 12 h increased the SP fraction to 7.8%, treatment with 20 µM CYA, a combination of CYA and PTX 24 h after removal of PTX for a similar time decreased SP fraction to ∼ 2% (Fig. 2, panels D, E and F, respectively) (*p*<0.05 in all samples). In PC3-TXR cells, the SP fraction was markedly reduced following treatment with CYA (Fig. 3C) or CYA and PTX combination (Fig. 3D). Real time RT PCR analysis indicates higher expression of pluripotency markers OCT4 and NANOG, and cancer stem cell markers CD133 and ALDH1 in SP fractions compared to non-SP (NSP) fractions in both DU145-TXR and PC3-TXR cells. Post-differentiation fate of SP cells was studied by real time RT-PCR and Hoechst staining after their isolation and re-culturing. These cells differentiated into a mix population comprising SP and NSP fractions which differed in their phenotype (Fig. 4, panels A and B, respectively). Expression analysis of post-differentiation mixed-populations indicates reduced transcripts of the ABC-transporter and cancer stemness marker ABCG2 and higher expression of cell proliferation marker minichromosome maintenance 7 (MCM7) compared to undifferentiated SP-fractions (Fig. 4D). Further treatment of post-differentiation SP populations with 25 µM CYA for 48 h resulted in the SP fraction significantly decreasing from 15.8% (panel E) to 0.6% (panel F, *p*<0.05). Table 1 shows the cell cycle analysis of the flow sorted SP and NSP cells. It was observed that, 62% NSP cells were in the G0-G1 and 30% in S phase in contrast to 71.5% and 21% for SP cells, respectively (*p*<0.05).

### Gene and Protein Expression in DU 145-TXR Cells

Expression of P-glycoprotein (P-gp) was assessed by Western blotting. Proteins were separated by SDS-PAGE and probed with anti-P-gp antibody to determine changes in protein expression following treatments. Figure 5A indicates that treatment with 25 µM CYA or a combination of CYA (10 µM) and PTX (0.5 µM) were equally effective, while treatment with PTX (0.5 µM) alone was ineffective. Expression of GLI-1 (Hh-pathway marker), OCT-4 (pluripotency/stem cell marker) and CD133 (cancer stem cell marker) was assessed using real time RT-PCR method and calculating the fold changes with respect to the Cp values obtained in DU145-TXR cells. Figure 5B show an increased expression of GLI-1, OCT-4 and CD133 (p<0.05 for all genes compared to DU 145 cells).

### Profiling of Prostate Cancer Cells Identifies miRNAs Altered in Chemoresistance

Recent studies have implicated various miRNAs such as let 7, miR 34a and miR 200c in regulating tumorigenesis and chemoresistance in humans [12], [15]. To establish whether chemoresistance to PTX in prostate cancer was regulated at the level of miRNAs, we studied the differential expression of several miRNAs known to be involved in various human cancers. The expression of eighty of the most common cancer-related miRNAs was simultaneously determined by a real time PCR method by adding template cDNA prepared from DU145, DU145-TXR as well as PC3 and PC3-TXR cells to 96-well plates containing miRNA specific primers. A ΔΔCt method was used to calculate fold changes using PTX-sensitive parental cells as controls. Figure 6A and B identifies the miRNAs whose expression is different between PTX-resistant and sensitive prostate cancer cells. As can be seen, several miRNAs such as 1, 18a, 138, 29b, 200c, 34a and 126 were downregulated while 193b, 30c, 155, 146a, 10b, 10a, 17, 125b, 373, 144 and 23b were upregulated in PTX-resistant cells with respect to parental cells. To confirm that our findings using this array methodology were accurate and that we were not reporting false positives, we validated our data by carrying out miScript primer assays on a select few of the differentially expressed miRNAs identified using the PCR array. Figure 6C lists the miRNAs used for data validation. As can be seen, a very high degree of concordance exists between the fold changes obtained through the array data and the validating primer assays. This suggests that our methodology for sample handling and data processing is correct and this increases our confidence in the validity of our approach.

To confirm that combination therapy is effective in targeting miRNAs involved in modulating chemoresistance, DU145-TXR cells were treated with a combination of PTX (0.5 µM) and CYA (10 µM) for 48 h and expression of miRs 200c and 34a was determined. Figure 6D demonstrates that combination therapy at concentrations effective in reducing cell viability and inducing apoptosis in PTX-resistant prostate cancer cells also upregulated two anticancer miRNAs involved in decreasing the spread of prostate cancer (34a) and increasing sensitivity to drugs like PTX (200c). While expression of miRNA 200c increased approximately 4 fold after treatment, there was nearly a 3-fold increase in miRNA 34a expression (*p*<0.05 compared to untreated control cells).

### Expression Analysis of Gene Targets of miRNAs 200c and 34a

Since miRNAs act by either repressing or cleaving their target mRNAs, we carried out gene expression analysis of several known downstream targets of several miRNAs, including miR200c and 34a identified in our cell and tissue miRNA arrays. Figure 7 delineates these target genes and their expression patterns in DU145-TXR cells compared to DU145 cells. As can be seen, there is upregulation of miR 200c target genes like VIMENTIN (2.5 fold), TUBB-3 (5 fold), ZEB 1 (5 fold), and miR 34a targets like CD44 (6 fold) and SIRT1 (2.5 fold) while the expression of another miR200c target E-CADHERIN was downregulated in DU145-TXR cells. In addition, gene targets of several miRNAs differentially expressed in this study were altered in DU145-TXR cells as well. These include DNMT1 (1.5 fold), BAK1 (2.4 fold) and PUMA (2.5 fold) downregulation.

### Confirmation of Cell Culture Studies in Clinical Samples

We also carried out SP fraction analysis and cancer miRNA profiling in clinical prostate tissues. SP fraction was analyzed by Hoechst staining as described earlier. Nearly 1% of total viable cells in prostate cancer tissue were SP cells (Fig. 8A), closely mirroring our findings in PTX-resistant prostate cancer cell lines. miRNA profiling of cancer tissue from a second patient was also carried out. For data analysis and subsequent validation, we used benign prostate tissue from a third patient biopsy as a normalization control. Similar to our results described earlier, we were able to identify several miRNAs such as miRs 17, let71, 31a, 193b, 29b, 155, let 7d, 9, 125b and 30 as being upregulated while miRs 145, 205, 34a, 126, 200c, 27b, 99a and 152 were downregulated in prostate cancer tissue (Fig. 8B). Further, there are a number of miRNAs that are common to our tissue and cell arrays in the present study. For example, not only are miRs 155, 29b, 34a, 200c, 193b and 17 differentially expressed in cell line miRNA analysis, their expression patterns too match those seen in the tissue arrays, thereby further validating our data obtained from chemoresistant cancer cells.

## Discussion

Advanced prostate cancer is usually treated with drugs like paclitaxel and docetaxel which eradicate the bulk of cells within the tumor. This therapeutic regimen often leads to chemoresistance for which few treatment strategies currently exist. Most tumors are heterogeneous and are composed of bulk cancer cells and a small population of pluripotent stem cells that are capable of self-renewal, are chemoresistant and capable of maintaining the tumor [5], [7]. These cancer stem cells (CSCs) are the reason why most chemotherapy drugs eventually lose their efficacy. We have recently shown that combining the antimitotic drug PTX with an Her-2 inhibitor, such as lapatinib can reverse chemoresistance [22]. There is some evidence that lapatinib exerts its anticancer effects in part by targeting the niche population of CSCs [24].

In recent years, inhibitors of Hh pathway such as CYA have been investigated for treating various malignancies as dysregulation of this pathway is a key step in generation and sustainment of cancers [17], [19], [20]. In prostate cancer too, there is evidence that Hh-pathway is upregulated [25]. Androgen independent prostate cancer cells such as DU145 and PC-3 have higher expression of Hh pathway members than androgen-dependent cells such as LnCaP [26]. Higher Gli-1 gene expression in PTX resistant DU145-TXR cells (Fig. 6) was observed compared to non-resistant parental cells. This prompted us to study the effect of combining CYA with PTX to treat the PTX chemoresistant. Treatment of DU145-TXR cells with 0.5 µM PTX and 10 µM CYA reduced cellular proliferation and cell viability while apoptosis, as measured by AnnexinV-FITC staining, increased significantly after 48 h of treatment (Fig. 1). In accordance with our previous observations [22], 0.5 µM PTX alone was not effective in reducing viability of DU145-TXR cells or inducing significant levels of apoptosis. Even though CYA at 25 µM decreased cell viability significantly, this reduction was still less than that achieved with lower concentration of drugs used in combination therapy. This ability to decrease proliferation of cancer cells while utilizing significantly reduced amounts of highly toxic drugs is a potential advantage for improved chemotherapy.

Many prostate cancers relapse due to multi-drug resistance (MDR) caused by the over-expression of ATP-binding cassette transporter proteins. These transporters actively efflux chemotherapeutic drugs from tumor cells and decrease the intracellular drug concentration [8], [27]. Therefore, modulation of MDR transporters is a promising approach to overcome MDR [28], [29], [30]. We have determined the effect of monotherapy with PTX or CYA, and their combination on the P-gp protein expression which is one of the major efflux pump systems involved in chemoresistance [31], [32]. Figure 5 indicates that treatment with a combination of PTX and CYA (0.5 µM and 10 µM, respectively) was able to downregulate P-gp protein expression. Thus, supplementing PTX with CYA can eliminate the chemoresistance of androgen independent prostate cancer cells by possibly suppressing MDR and decreasing P-gp expression.

In addition to the P-gp system, chemoresistance is regulated by various other mechanisms, including cancer stem cells which are highly chemoresistant due to their ability to efflux chemotherapy drugs. Many current techniques used for characterization of these stem cells rely on the presence of cell surface markers such as CD133 and CD44 which were used to isolate these cells using a flow cytometry approach [5], [33]. However, this approach suffers from the fact that during processing, including trypsinization, many of these surface markers are cleaved and therefore not available for antibody binding. Further, CD 133 is not a reliable marker for isolating CSCs as its expression may not be restricted to cancer stem cells and that even CD 133^−^ cells can initiate tumorigenesis [34]. For this reason, the vital dye Hoechst 33242 was used in the present study for separating SP from main population of cancer cells. The SP fractions are a subpopulation of CSCs which were first identified in a hematopoietic stem cell isolation procedure [9]. These cells have a unique fluorescence-activated cell sorting (FACS) signature and can be separated by a flow cytometer with a UV laser as they are distinct from cells that take up the Hoechst 33342 dye. The SP fraction is capable of sustained expansion *ex vivo* and can generate both SP and non-SP progeny. To understand the mechanism of chemoresistance in chemoresistant prostate cancer cells, we hypothesized that these cells are likely to contain a higher percentage of cells that are chemoresistant and therefore have increased ability to efflux chemotherapy drugs like PTX. Staining with Hoechst dye resulted in a creation of a typical flow cytometric dot plot profile where P4 gated region in Fig. 3 and P8 gated region in Fig. 4 is lightly stained compared to other cells. These lightly stained cells are the SP fraction which constitutes approximately 2–4% of total viable cells. In contrast, the parental PTX-sensitive DU145 cells had only 0.2% cells in the P4 region, thereby confirming our hypothesis of a distinct cell population with the ability to efflux hydrophobic drugs effectively, thereby inducing chemoresistance. Treatment with verapamil, an inhibitor of calcium gated channels, and known suppressor of cancer stem cells [35] significantly reduced SP fraction thereby confirming that the population being gated is indeed related to CSCs. It is a known fact that in relapsed tumors and metastatic cancers, there are an increased percentage of cancer stem cells and SP fractions. In the present case also, treatment of DU145-TXR cells with 1 µM PTX actually increased SP fraction to nearly twice that observed in untreated samples (7.7% vs 3.4%). These observations are supported by several lines of evidence that indicate the role played by Hh-pathway in maintaining stem cells, including cancer stem cells in various tumors [36], [37]. Further, treatment with Hh-inhibitors can reduce or eliminate tumorigenic stem cells [37], [38], [39]. The present data indicates that there is more expression of OCT 4, a marker for self-renewal and pluripotency and CD133, a general marker for cancer stem cells [5] in DU145-TXR cells. This emphasizes the potential reservoir of CSC like cells mixed with bulk cancer cells in the chemoresistant cell line. From the theory of biogenesis of CSCs, it is clear that SP fraction have the potential for asymmetric differentiation and can give rise to non-SP fractions which will go on to form the bulk tumor cells [5], [40]. To verify this theory in prostate cancer cells, pure SP and NSP fractions were isolated by flow sorting recultured and photographed. Fig. 4A and B are representative micrographs of isolated SP cells and NSP fractions. As can be seen, SP fraction has more elongated cells with typical fibroblastic phenotype while NSP cells were more rounded. Further, compared to the non-SP, the SP cells from PTX-resistant versions of both DU 145 and PC3 cells had higher gene expression of pluripotency markers OCT4 and NANOG [41] and increased expressions of cancer stem cell markers ALDH1 and CD133 (Fig. 4C). This is not surprising since CSCs, including SP fractions are capable of self-renewal and can therefore persist in cultures such as those established after flow-sorting. Comparison of expression of ABCG2 and MCM7 transcripts also differed significantly between SP fraction and their progeny mixed populations. While expression of efflux pump ABCG2 was higher in SP cells, proliferation marker MCM7 [42] was highly expressed in post-differentiated cells compared to initial SP cells suggesting the propensity of these cells for reduced proliferation. Even after differentiation, the number of SP-fraction cells was very high (almost 16%) which vanished after treatment with a high concentration of CYA (25 µM) for 48 h suggesting that suppression of Hh-pathway may be playing a role in their persistence and maintenance (Fig. 4E and F, respectively). Isolated SP and NSP fractions were also used to confirm the cell cycle stages. We have shown that more SP cells were in G0/G1 phase than non-SP cells (Table 1). This is in accordance with our gene expression data and studies from other workers who have demonstrated that the inherent stem cell like properties of SP population include a propensity for quiescent phase [21], [40]. This may be the reason CSCs can bypass the G1/S checkpoint and thus maintain their ability for self-renewal and remain pluripotent.

Recent studies have demonstrated that dysregulation of various miRNAs is associated with the etiology, metastasis and prognosis of various cancers including prostate cancer [13], [14]. So far, nearly 50 miRNAs have been reported to be significantly expressed in human prostate cancer but only a few have been linked directly to the pathology and progression of prostate cancer [43], [44]. To confirm the hypothesis that chemoresistance to PTX involves differential expression of miRNAs, a focused profiling of miRNAs known to be involved in various malignancies was carried out in DU145-TXR and PC3-TXR cells (Fig. 6A and B, respectively). Several miRNAs involved in prostate cancer and other malignancies were differentially expressed in chemoresistant prostate cancer cells. Significant among these include miR200c, miR34a and miR29b, all of which are downregulated in DU145-TXR cells. These data trends are in accordance with studies in literature which shows that miR34 is downregulated in prostate cancer and its experimental replenishment in cultured cells prevents metastasis and invasion [45]. miRNA200c, which is downregulated in DU145-TXR cells, is a tumor-suppressor miRNA known which maintains ‘epithelialness’ of cancer cells by preventing EMT and the assumption of an aggressive chemoresistant mesenchymal phenotype. A recent study by Cochrane et al. confirmed reduced expression of miR200c as being responsible for chemoresistance to microtubule stabilizers (such as taxanes) in endometrial, breast and ovarian cancer cells. In multi-drug resistant cancer cells, restoration of miR200c levels by transfection of its mimic restored chemosensitivity to PTX [15]. This observation is further supported by evidence that the loss of miR200c expression results in a highly aggressive, mesenchymal and chemoresistant phenotype in lung cancer cells [46]. In view of the findings described above, we decided to confirm the effect of our combination therapy on the expression of miR200c and miR34a (Fig. 6D). After treatment with 0.5 µM PTX and 10 µM CYA for 48 h, the expression of both miRs 200c and 34a were increased significantly, compared to non-treated TXR cells. It is therefore feasible that this increase in the levels of these miRNAs due to the combination therapy is responsible for the increased apoptosis and decreased cell proliferation (Fig. 1).

Expression analysis of select genes which are known targets of differentially expressed miRNAs, including miR200c and 34a was carried out to further support our miRNA data. [47]. Figure 7 details some miRNA target genes which are altered in DU145-TXR cells compared to the parental DU145 cells. SIRT-1 is suppressed by miR34a and is upregulated in prostate cancers following loss or downregulation of miR34a [48]. In wild type cells, downregulation of SIRT-1 by miR34a results in increased apoptosis [49]. This suggests that the greater resilience of TXR cells may be due this loss of miR34a. CD 44 is also a surface marker for CSCs [5] and it stands to reason that it will be upregulated in a cell line where miR34a is downregulated and where SP fraction cells are found in higher numbers. Downregulation of miRNA200c leads to an EMT transformation which is characterized by a loss of E-CADHERIN (E-CAD) and upregulation of VIMENTIN (VIM) [15]. In aggressively metastatic cell lines from breast, ovarian and endometrial cancers, E-CAD levels are suppressed by ZEB-1 which is upregulated as a result of suppression of miR200c. Downregulation of miR200c also increases the protein and gene levels of TUBB-3. It has been suggested that decreased protein levels of TUBB-3 protein correlate to increased cell death in response to microtubule-targeting agents such as PTX. DNMTa1 which is also upregulated is a target of miR29b and is regulated by global hypomethylation. Loss of miR29b results in upregulation of DNMTa1 at protein and gene levels in aggressive cancer cells [50] such as seen in the present study. Bak-1 which is downregulated in DU145-TXR cells is a target of miR125b, a known oncomir which confers chemoresistance to PTX in breast cancer cells through suppression of Bak-1 gene [51]. Another target of miR125b is PUMA which is a proapoptotic gene and is suppressed when miR125b is overexpressed in cancer cells [52].

Finally, to correlate and extrapolate the above findings to clinical studies, SP fraction analysis (Fig. 8A) and miRNA profiling of prostate cancer tissues (Fig. 8B) was performed. Presence of distinct SP fractions in clinical prostate cancer tissue is supportive of our cell culture data. Further, as several miRNAs which are verifiably altered in PTX-resistant cell lines, such as miR200c and miR34a, are similarly modified in a clinical prostate cancer sample, our hypothesis and the overall approach taken to confirm it are strengthened substantially

### Conclusion

We have established that chemoresistance to PTX in DU145-TXR and PC3-TXR cells is possibly regulated by miRNAs which are differentially expressed when the PTX sensitive cell line is transformed to a resistant phenotype. miRNAs are known to regulate cancer stem cells, chemoresistance and EMT since CSCs possess a mesenchymal phenotype that allows them to be invasive. This crosstalk between miRNAs, cancer stem cells and the resulting physiologic processes such as metastasis and chemoresistance is explained in the schematic in Figure 9.

The present study thus suggests a strategy to restore chemosensitivity to drug-resistant cancer cells, thereby ensuring that cancers do not relapse due to generation of CSCs in tumors.
